# Genes Involved in the Production of Antimetabolite Toxins by *Pseudomonas syringae* Pathovars

**DOI:** 10.3390/genes2030640

**Published:** 2011-09-15

**Authors:** Eva Arrebola, Francisco M Cazorla, Alejandro Pérez-García, Antonio de Vicente

**Affiliations:** 1 Experimental Station La Mayora, Institute of Subtropical Horticulture and Mediterranean “La Mayora” (IHSM-UMA-CSIC), Algarrobo-Costa (Málaga) 29750, Spain; 2 Microbiology Department, Institute of Subtropical Horticultural and Mediterranean “La Mayora” (IHSM-UMA-CSIC), Faculty of Science, University of Málaga, Associated Unit with the CSIC, Campus de Teatinos, Málaga 29071, Spain; E-Mails: cazorla@uma.es (F.M.C.); aperez@uma.es (A.P-G.); adevicente@uma.es (A.dV.)

**Keywords:** secondary metabolite, gene cluster, tabtoxin, phaseolotoxin, mangotoxin, phytotoxin

## Abstract

*Pseudomonas syringae* is pathogenic in a wide variety of plants, causing diseases with economic impacts. *Pseudomonas syringae* pathovars produce several toxins that can function as virulence factors and contribute to disease symptoms. These virulence factors include antimetabolite toxins, such as tabtoxin, phaseolotoxin and mangotoxin, which target enzymes in the pathways of amino acid metabolism. The antimetabolite toxins are generally located in gene clusters present in the flexible genomes of specific strains. These gene clusters are typically present in blocks of genes that appear to be integrated into specific sites in the *P. syringae* core genome. A general overview of the genetic organization and biosynthetic and regulatory functions of these genetic traits of the antimetabolite toxins will be given in the present work.

## 1. Introduction

*Pseudomonas syringae* is an opportunistic phytopathogenic bacterium that normally exists as an epiphyte, but if the host's defences are compromised, *P. syringae* becomes pathogenic. This species of bacteria infects a large variety of plants and produces a wide spectrum of phytotoxic compounds [1], and it is well adapted to survive in plant environments. The phytotoxins produced by *P. syringae* pathovars are not host-specific [2]. Although not essential for pathogenicity, they generally act as virulence factors and are involved in the production of disease symptoms in many plants [1,3]. Some of the most important phytotoxins produced by *P. syringae* are toxins belonging to the syringomycin group, which is a virulence determinant of phytopathogenicity. Syringomycin toxins consist of a polar peptide head with a lipophilic, fatty-acid tail; they have amphiphilic properties, which can lower surface tension and interact with and alter the integrity of cellular membranes [4,5]. The genes involved in the production of these toxins, including the non-ribosomal peptide synthetase (NRPS) systems, are mainly clustered in the bacterial chromosome [1,6]. For example, the syringomycin (*syr*) gene cluster of *P. syringae* pv. *syringae* strain B301D is about 37 kb in size, and it consists of two non-ribosomal peptide synthetases (NRPS; *syrB1* and *syrE*); the syringopeptin gene cluster (*syp*), which is directly adjacent to the *syr* gene cluster, is 74 kb and carries 22 NRPS modules [7]. Another phytotoxin produced by several pathovars of *P. syringae* is coronatine. This virulence factor promotes entry of the bacteria into the plant host by stimulating the opening of the stomata [8] and suppressing the salicylic acid-dependent host defences [9,10]. Several adjacent gene clusters are necessary for the biosynthesis of coronatine, including the *cor* cluster (*corPSR*), which helps control the transcription of the *cma* and *cfa* clusters [11]. According to results obtained from the study of genes belonging to this cluster and are responsible for the biosynthesis of coronamic acid, which is non-proteinogenic, the involvement of a thiotemplate mechanism that could be located in the *cma* cluster is likely [12,13]. The polyketide portion of coronatine, coronafacic acid, is synthesized by the *cfa* cluster, which consists of nine open reading frames [14,15].

**Figure 1:**
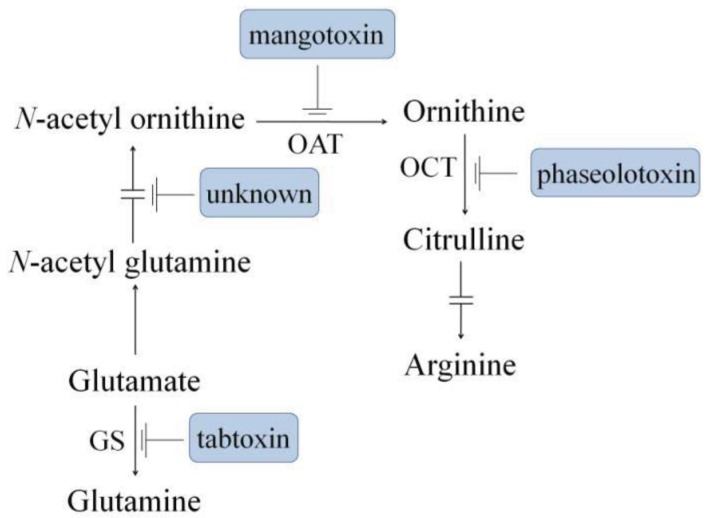
Schematic representation of glutamine and arginine biosynthetic pathway and the corresponding target enzymes inhibited by antimetabolite toxins produced by different *Pseudomonas syringae* pathovars. Unknown refers to the uncharacterized toxic activity from some strains of *P. syringae* pv. *tomato*. GS: Glutamine synthetase; OCT: Ornithine carbamoyltransferase; OAT: Ornithine acetyl transferase. Figure partially adapted from Arrebola *et al.* [61].

The antimetabolite phytotoxins, which are the subject of this overview, consist of small peptide molecules that interfere with the nitrogen metabolism of host cells and influence the course of disease development or symptoms. Although the antimetabolite toxins are not required for *P. syringae* to be pathogenic, they generally function as virulence factors, and their production results in increased disease severity [3,16,17]. The targets of all the described antimetabolite toxins are enzymes involved in the biosynthetic pathways of amino acids, such as glutamine or arginine. One of the better known antimetabolite toxins is tabtoxin, which is a β-lactam that inhibits glutamine synthetase (GS) (Figure 1), causing a glutamine deficit and an ammonium increment. Another well-known antimetabolite toxin is phaseolotoxin, which is a sulfodiaminophosphinyl peptide that disrupts the urea cycle by inhibiting ornithine carbamoyltransferase (OCT), causing arginine deficiencies. Mangotoxin, which is a more recently described toxin, inhibits ornithine acetyltransferase (OAT) (Figure 1), producing an ornithine deficit and interfering with arginine metabolism. Two other antimetabolite toxins, both of which inhibit the enzymes that catalyse the transformation of *N*-acetyl-glutamate into *N*-acetyl-ornithine, have also been detected. However, unstable precursors have hindered our ability to gain knowledge about the target enzymes of these last two antimetabolite toxins [18,19]. Studies on the phylogeny of antimetabolite toxins have concluded that toxin production is not irremediably linked to a specific pathovar or pathovars of *P. syringae*. Toxigenic and non-toxigenic *P. syringae* strains may belong to the same pathovar. Moreover, it is unusual to detect strains that produce more than one kind of antimetabolite toxin, so toxin production can be consider poorly associated with the host of isolation [20].

The genes encoding the antimetabolite toxins have not been found homologues outside of the *P. syringae* genome [20]. Study of the metabolism of toxins has led to the discovery of novel biosynthetic mechanisms, which typically involve non-ribosomal synthesis catalysed by multifunctional proteins or polypeptide complexes and intermediates that are transferred between enzymatic domains and not released into the cytoplasm. These enzymes are normally encoded in gene clusters, which are typically present in block of genes that appear to integrate into specific sites in the *P. syringae* core genome [7]. These gene clusters present qualities that are inconsistent with clonal (vertical) evolution. Contrarily, the evolution of toxin-producing genes is very likely driven by horizontal gene transfer [21].

The main goal of the current review is to present the research studies that have resulted in a better understanding of the genetic organization, biosynthetic pathways and regulatory functions of the three best-known antimetabolite toxins produced by pathovars of *P. syringae*.

## 2. Tabtoxin

**Figure 2:**
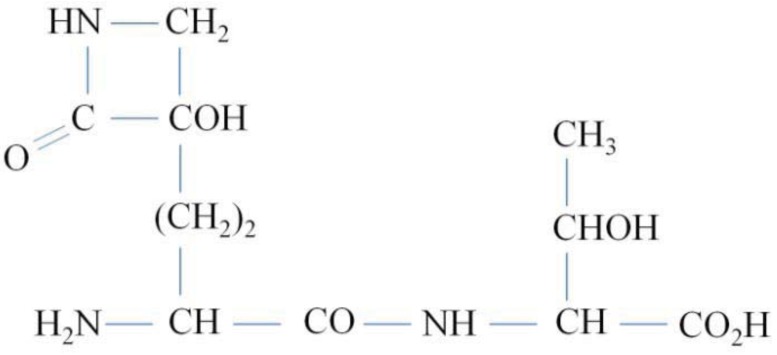
Chemical structure of tabtoxin.

Tabtoxin is a phytotoxic dipeptide produced by *P. syringae* pvs. *tabaci*, *coronafaciens* and *garcae* [22]. It is composed of tabtoxinine-β-lactam [2-amino-4-(3-hydroxy-2-oxoazacyclobutan-3-yl) butanoic acid] and either serine or threonine (Figure 2) [23]. It has been suggested that *in planta*, some peptidases convert tabtoxin to tabtoxinine-β-lactam (TβL), which irreversibly inhibits the target enzyme glutamine synthetase (GS). This inhibition results in the abnormal accumulation of ammonium, causing the characteristic chlorosis [24].

### 2.1. Resistance

The first detected gene related to tabtoxin was named the tabtoxin resistant gene (*ttr*); it is specific for the inactivation of tabtoxin and not for other inhibitors of glutamine synthetase, such as bialaphos and methionine sulfoximine [25]. To examine the function of the *ttr* gene, tabtoxin was incubated with [^14^C]-acetyl-CoA in crude bacterial extracts. Radioactive acetylated products were detected only in extracts of *Escherichia coli* transformed with a tabtoxin resistance gene, indicating that the *ttr* gene encodes an enzyme which acetylates tabtoxin or TβL [26]. In some works, it has been speculated that the acetylation probably occurs in a TβL amino group according to a mechanism similar to the inactivation of bialaphos [27,28]. To confirm this function of the *ttr* gene, Anzai *et al.* [26] transformed tobacco plants with the *ttr* gene, confirmed the integration of *ttr* into the genomic DNA of transgenic tobacco plants and tested its expression levels. When the transgenic tobacco plants were inoculated with *P. syringae* pv. *tabaci*, none of them produced the chlorotic halo typical of the wildfire disease seen in non-transgenic controls. This strongly suggested that the *ttr* gene expressed in tobacco plants provides resistance to the effects of tabtoxin [26]. Sometime later, the function of the *ttr* gene was confirmed, and the mode of action was described by analysis of the structure of the *ttr* gene product (TTR) to confirm that TTR is an enzyme that functions as an acetyl-transferase [29]. Tabtoxin-resistance protein TTR was determined to be an enzyme that renders tabtoxin-producing pathogens tolerant to their own phytotoxin.

Meanwhile, Kinscherf *et al.* [30] continued searching for genes related to tabtoxin biosynthesis. By constructing *Tn5*-defective mutants, these authors characterized a DNA region highly conserved among tabtoxin-producing pathovars of *P. syringae*, which could be deleted at a relatively high frequency (10^−3^/cfu), in the BR2 strain of *P. syringae*. The cosmid pRBL823, which was obtained from analysis of *Tn5* mutants, contains clones of about 32 kb of genomic DNA, and it can restore the production of tabtoxin in Tox^−^ spontaneous deletion mutants; the cosmid even enabled tabtoxin production and resistance in the *P. syringae* epiphyte Cit7, which does not naturally produce tabtoxin, indicating that pRTBL823 contains a complete set of genes for the biosynthesis of and resistance to tabtoxin. However, when sequences containing the *ttr* gene were compared with the cosmid pRTBL823 by Southern blot analysis, no cross-hybridisation was found. According to their data, the *ttr* gene is located outside the cloned region, which does not support the conclusion that this gene is the only source of resistance to TβL exhibited by tabtoxin-producing strains. Moreover, their results indicate that tabtoxin is required by the *P. syringae* BR2 strain for both chlorosis and lesion formation in beans. However, toxin production was not required for growth *in planta*; until then, it had been impossible to genetically separate toxin production from the disease symptoms caused by this strain of *P. syringae* pv. *tabaci* [30].

### 2.2. Identification of the Genes Involved in Tabtoxin Biosynthesis

**Figure 3:**
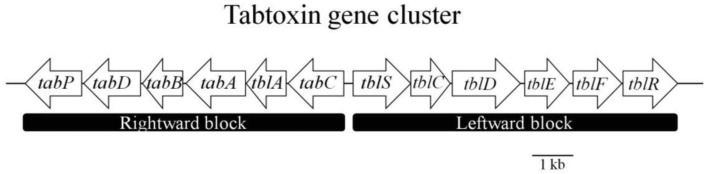
Structural organization of the tabtoxin biosynthetic gene cluster. The different coding sequences (CDs) are represented by arrows indicating the direction of transcription. The name of each gene is indicated in each arrow. Figure partially adapted from Gross and Loper [7].

The study of approximately 3 kb of the cloned region revealed the presence of the genes *tbl*A and *tab*A (also called *tbl*B), which are required for tabtoxin production (Figure 3). The *tblA* gene encodes a product with an amino acid sequence unrelated to those of known polypeptides. This gene seems to be regulated by the *lemA* (now named *gacS*) gene via mRNA. The gene *lemA* (*gacS*) belongs to a family of two-component regulatory genes (*gacA*/*gacS*) involved in pathogenesis [31]. The regulation of the *tblA* gene may occur at the level of transcription; however, posttranscriptional effects, such as message stability, have not been ruled out.

Barta *et al.* [31] had no evidences that the *tblA* gene was part of a multigenic operon; on the contrary, mutations in genes upstream of *tblA* did not inhibit production of its transcript, nor did they prevent tabtoxin production. Furthermore, operon fusion data suggested the presence of a fairly strong promoter separate from the *tblA* gene [33]. The *tabA* product presented an amino acid sequence homologous to *meso*-DAP decarboxylase (the *lysA* gene product). The similarity between *tabA* and *lysA* suggests that the biosynthetic pathways of lysine and TβL could be related, however, *tabA* was unable to complement an *Escherichia coli lysA* mutant [32] The *tabA* gene is located immediately downstream of the *tblA* locus; however, the 1 kb transcript was not large enough to encode the predicted gene products of both the *tblA* and *tabA* genes. This could indicate that these two genes are transcribed separately. Their studies also showed that bacteria in minimal medium produced the *tblA* message during all growth phases. The *tblA* transcript was also detected in rich medium; however, the presence of other amino acids interferes with the detection of TβL by both bioassay and amino acid analysis [33], which is a common problem in the study of antimetabolite toxins.

### 2.3. Biosynthesis Proposals

Liu and Shaw [34] attempted to clarify the biosynthetic pathway of TβL by obtaining mutants of the *dapB* gene from tabtoxin-producing strains. The *dapB* gene encodes l-2,3-dihydrodipicolinate (DHDPA) reductase, the second enzyme of the lysine branch of the aspartic amino acid family, and mutation of *dapB* produces diaminopimelate (DAP) auxotrophs and tabtoxin-negative mutants. DAP mutants were obtained from a parent strain (tabtoxin-producing strain) and the mutants in which the TβL biosynthetic locus was deleted. Restriction digestion and hybridisation analysis indicated that *Tn5* insertion had occurred in the same locus in both the mutants and the parents. These data led to the conclusion that in *in vitro* conditions, the wild-type, tabtoxin-producing strain contained a single functional copy of *dapB*, a gene that was not located in the TβL biosynthetic region. In addition, this region did not contain a gene that encodes a protein with DHDPA reductase activity. In addition to requiring DAP and lysine for growth, *dapB* mutants also failed to produce TβL, indicating that neither DAP nor lysine is a tabtoxin precursor [34]. Moreover, results obtained from *dapB* gene complementation, coupled with the observed phenotype in *dapB* mutants, lead to the prediction that DapB was an enzyme required for the biosynthesis of both DAP and TβL and that tetrahydropicolinate (THDPA) was a common intermediate in those biosynthetic pathways [34].

A few years later, Kinscherf and Willis [35] published a study that revealed the biosynthetic gene cluster for TβL in *Pseudomonas syringae*. They performed an analysis of the tabtoxin gene transfer block, where they observed that the portion of the tabtoxin gene island closest to the *lysC* tRNA gene contain genes associated with genetic dynamism; even the gene adjoining the tRNA gene encodes a full-length product with high similarity to XerC (tyrosine recombinase). Such genes have often been found near the beginning of chromosome regions thought to result from the horizontal transfer of DNA into the current host [36]. The biosynthetic gene cluster contains the “leftward” block, which is separated from the “rightward” block by a 1 kb, AT-rich section that could be a bidirectional promoter (Figure 3, Table 1). The first gene product in the left biosynthetic block, as transcribed right to left, is TabC, which contains a motif homologous to some zinc-binding proteins but was otherwise novel in the database. The next gene product is TblA, which has weak similarity to methylase but was also novel, then TabA, which has high similarity to diamopimelate decarboxylase (LysA). TabB was highly similar to succinyldiaminopimelate aminotransferase (DapD), and TabD was an Aat-like aminotransferase. Finally, TabP is a putative zinc metallopeptidase that seems to be responsible for the maturation event that converts tabtoxin to its toxic form, tabtoxinine-β-lactam.

The first two genes in the right biosynthetic block (Figure 3, Table 1) encode proteins similar to a pair known to be involved in the synthesis of clavulanic acid. The first open reading frame (ORF) described, TblS, is similar to β-lactam synthase and is related to the asparagine synthetases that catalyse the formation of the β-lactam ring in clavulanic acid [37]. The adjacent ORF, TblC, resembles clavaminic acid synthase. TblD appears to represent an interesting gene fusion whose product is a full-length glucose-methanol-choline-oxidoreductase (GMC-oxidoreductase) with a GNAT family acetyltransferase domain on the carboxy-terminus. TblE is a putative membrane protein, and TblF, is presumed to be an enzyme with similarity to d-alanine-d-alanine ligase. The last ORF, which encodes a member of the transporter and multi-drug resistance, was designated as TblR [35]. Thus, tabtoxin shares part of the biosynthetic pathway of lysine (*dabABCDE*), branching off after tetrahydrodipicolinate but before diaminopimelate formation. From this branch point, tabtoxin is assembled by the actions of TblS, TblC, TblD and TblE in conjunction with TblF. Upon assembly, the completed tabtoxin can be converted by the metallopeptidase TabP into the toxin TβL, which is subsequently exported by the transporter TblR [7].

Knowledge of the gene cluster responsible for the biosynthesis of tabtoxin has allowed the development of other techniques and studies. For example, specific detection methods for tabtoxin-producing pathovars have been developed, as have PCR protocols that utilize oligonucleotide primers derived from the coding sequences of the *tblA* and *tabA* genes for the quick and reliable detection of tabtoxin-producing bacteria. This PCR detection method uses the primer sets to amplify a single 829-bp amplification product of *tblA* and another single 1020-bp amplification product of *tabA*, genes that proved to be specific for tabtoxin-producing organisms [38].

**Table 1:** Tabtoxin biosynthetic genes identified from *Pseudomonas syringae* pv. *tabaci* BR2 and deposited in National Center for Biotechnology Information (NCBI) with accession code DQ187985.

**Genes**	**Putative function**	**NCBI gene id number**
*tabC*	Putative zinc binding protein	AF519896.1 a
*tblA*	Tabtoxin biosynthesis enzyme	AF519896.1
*tabA*	Meso-DAP decarboxylase	AF519896.1
*tabB*	Succinyldiaminopimelate aminotransferase	M88485
*tabD*	Aat-like aminotransferase	AY091643.1
*tabP*	Zinc metallopeptidase	AY083468.1
*tblS*	β-lactam synthase	AF521701.1
*tblC*	Similar to clavaminic acid synthase	AY220496.1
*tblD*	GMC-oxidoreductase	AY744151.1
*tblE*	Putative membrane protein	AY254168.1
*tblF*	D-Ala-D-Ala ligase	nd b
*tblR*	Multidrug resistance transporter protein	AY254170.1

a *tabC*, *tblA* and *tabA* genes are annotated together with the same accession number;

b *tblF* has not found in NCBI database.

## 3. Phaseolotoxin

**Figure 4:**
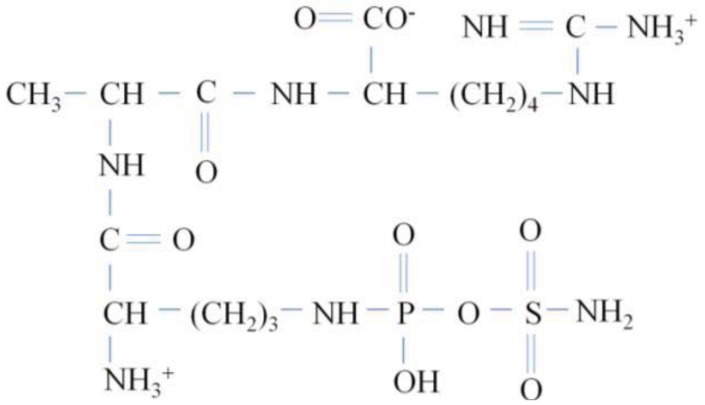
Chemical structure of phaseolotoxin.

Phaseolotoxin is a phytotoxic tripeptide consisting of ornithine, alanine and homoarginine that is linked to an inorganic sulfodiaminophosphinyl moiety (Figure 4) [16,39]. Phaseolotoxin is produced at relatively low temperatures (18 to 22 °C) by *Pseudomonas syringae* pvs. *phaseolicola* and *actinidiae*, pathogens causing halo blight in beans and canker in kiwi fruit, respectively. It is also produced by *P. syringae* pv. *syringae* CFBP3388 [40]. Phaseolotoxin can be cleft by peptidases and can liberate sulfodiaminophosphinyl ornithine (Psorn, also called octicidine [39]), the irreversible inhibitor of ornithine carbamoyltransferase (OCT), an enzyme that catalyses the conversion of ornithine to citruline.

### 3.1. Resistance

**Figure 5:**
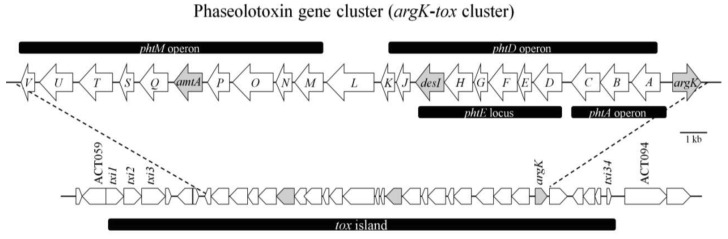
Structural organization of the phaseolotoxin biosynthetic gene cluster (*pht* cluster). The different CDs are represented by arrows indicating the direction of transcription. The grey arrows indicate the principal genes identified and characterized by researchers. The different loci and operons are indicated by black bands. The *pht* cluster is located in the *tox* island corresponding to the phaseolotoxin genomic island. The genomic map of the *tox* island and the regions bordering it are shown. Figure partially adapted from Genka *et al.* [55] and Aguilera *et al.* [49].

Initial studies on genes involved in the biosynthesis of phaseolotoxin using random mutagenesis by *Tn5* insertion. This resulted in the localization of a *Kpn*I genomic fragment of about 28 kb (including the *Tn5* insertion), which restores the production of phaseolotoxin in defective *Tn5* mutants. The pattern of restoration observed in the mutants and their plasmids evoked a cluster of genes initially called *tox* [41]. The first gene identified in the phaseolotoxin gene cluster was *argK* (Figure 5), which encodes a phaseolotoxin-resistant ornithine carbamoyltransferase (ROCT), which prevents PSorn inhibition [42]. Hatziloukas and Panopoulos [42] conducted a study in which they established that the enzyme ROCT did not evolve from the housekeeping sensitive OCT (SOCT). This was consistent with the idea that *argK* is not derived from the *argF* gene, which encodes SOCT, of *P. syringae* pv. *Phaseolicola*. The *argK* gene must have immigrated into the organism's genome from another source by horizontal transference. The physical proximity of *argK* to the cluster of the genes involved in the biosynthesis of phaseolotoxin and its role in phaseolotoxin-resistance suggest that the entire *argK-tox* cluster was transferred en masse into the *P. syringae* pv. *phaseolicola* genome [42].

The *argK* transcription and regulation studies performed by Hatziloukas and Panopoulos revealed three *argK*-specific transcripts and a transcription start point near the putative ROCT initiation codon. The results of RNA sizing indicated that a small (presumably monocistronic) *argK* mRNA and two larger (potentially bi- or multicistronic) *argK* mRNAs exist in the cells (Figure 5). The *argK* promoter was stimulated in arginine-supplemented cultures at both high and low temperatures, but to a different degree. The greater activation of the *argK* promoter at 28 °C effectively cancelled the apparent repression of the *argK* promoter by elevated temperature in the minimal medium [42].

### 3.2. Identification of Genes Involved in Phaseolotoxin Biosynthesis

One year later, in 1993, *Zhang et al.* [43] published a study on the genetic organization of a cluster of genes involved in the production of phaseolotoxin. They showed that *P. syringae* pv. *phaseolicola* genomic clone pHK120, which complemented all their Tox^−^ mutants, can be divided into at least eight complementation groups, *phtA* to *phtH* (Figure 5). Although pHK120 contains most of the genes involved in the production of phaseolotoxin, they concluded that it does not contain all of them, since pHK120 does not contain the ROCT gene (*argK*). The fact that transconjugants containing pHK120 produced toxin at 28 °C indicated that the thermoregulation of toxin production was overridden by pHK120. The plasmid may have contained an activator; overproduction of this activator could abolish thermoregulation. Another possible explanation is that some or all of the phaseolotoxin genes present in pHK120 harbour DNA binding sites which titrate the putative repressor protein produced by *P. syringae* pv. *phaseolicola* at 28 °C. This clone does not harbour a structural or regulatory gene involved in toxin production, but it contains DNA-binding motifs similar to those involved in binding proteins in other systems. On the basis of these results, the authors proposed that when present in multiple copies, the fragment harbouring DNA-binding sites titrates a repressor protein produced at 28 °C, thereby allowing toxin biosynthesis to occur [43,44]. Similar DNA-binding motifs were found in the upstream region of the *argK* gene, which is co-ordinately regulated with phaseolotoxin genes by temperature [42].

Based on what we now know, the production of phaseolotoxin occurs at low growth temperatures (18–22 °C) and is genetically controlled by a group of genes called *tox* according Peet *et al.* [41] or *pht* according to Zhang *et al.* [43] clustered in a region approximately 28 kb in size. The phaseolotoxin biosynthetic cluster is now called the *argK-tox* cluster because it includes *argK* [45].

The fatty acids desaturase gene (*desI*) (Figure 5, Table 2) was unambiguously identified and located at the 3′-end of the complementation group *phtE* in the middle of the *argK-tox* cluster. Genes placed downstream from *desI* were revealed, as *desI* spans a region essential for phaseolotoxin production. The link between the *desI* product and the biosynthesis of phaseolotoxin is not immediately obvious; however, if it is possible to establish a relationship between fatty acids desaturase, phaseolotoxin secretion at low temperatures and cell membrane fluidity [45], then desaturase action produces an increase in membrane fluidity to facilitate the phaseolotoxin secretion. The next gene identified in the *argK-tox* cluster was called *amtA* (Figure 5, Table 2), which encodes amidinotransferase. In fact, biochemical activity corresponding to an amidinotransferase was detected in *P. syringae* pv. *phaseolicola* extracts in previous studies [46,47]. The amidinotransferase activity detected was shown to catalyse the transfer of an amidino group from arginine to lysine to produce one molecule of homoarginine and one molecule of ornithine, both of which are precursors in the biosynthesis of phaseolotoxin. Mutants in *amtA*, defective in amidinotransferase activity, lost the ability to synthesize homoarginine and phaseolotoxin when grown in minimal medium at 18 °C [48]. According the results obtained by these authors, *amtA* was thermoregulated following a pattern similar to that obtained for *argK*.

**Table 2:** Phaseolotoxin genes identified from *Pseudomonas syringae* pv. *phaseolicola* NPS3121 and its annotation in National Center for Biotechnology Information (NCBI) with accession number DQ141263.1.

***Ps.phaseolicola***	**NPS3121**	**Putative function**	**Ortholog genes** a		
	
**Genes**	**NCBI annotation**		***Ps.phaseolicola*** **1448A**	***Ps.phaseolicola*** **MAFF302282**	***Ps.actinidiae*** **Kw11**
*argK*	*argK*	Phaseolotoxin insensitive ornithine carbamoyltransferase	PSPPH4319	*argK*	*argK*
*phtA*	*ptx2*	Hypothetical protein	PSPPH4318	*txi28*	*tx*i*28*
*phtB*	*ptx3*	Hypothetical protein	PSPPH4317	*tx*i*27*	*tx*i*27*
*phtC*	*ptx4*	Hypothetical protein	PSPPH4316	*tx*i*26*	*tx*i*26*
*phtD*	*ptx5*	Hypothetical protein	PSPPH4315	*tx*i*25*	*tx*i*25*
*phtE*	*ptx6*	Hypothetical protein	PSPPH4314	*tx*i*24*	*tx*i*24*
*phtF* (*argD*)	*ptx7*	Ornithine aminotransferase	PSPPH4313	*tx*i*23*	*tx*i*23*
*phtG*	*ptx8*	Hypothetical protein	PSPPH4312	*tx*i*22*	*tx*i*22*
*phtH*	*ptx9*	Fatty acid desaturase	PSPPH4311	*tx*i*21*	*tx*i*21*
*desI*	*desA*	Fatty acid desaturase	PSPPH4310	*tx*i*20*	*tx*i*20*
*phtJ*	*ptx11*	Deoxycytidine triphosphate deaminase	PSPPH4309	*txi19*	*txi19*
*phtK* (*dcd*)	*ptx12*	Deoxycytidine triphosphate deaminase	PSPPH4308	*txi*19- *txi*18	*txi*19- *txi*18
*phtL*	*ptx13*	Pyruvate phosphate dikinase PEP	PSPPH4307	*txi17*	*txi17*
*phtM*	*ptx*14	Hypothetical protein	PSPPH4306	*txi15*	*txi15*
*phtN* and *phtO*	*ptx15*	Hypothetical protein	PSPPH4305	*txi14*	*txi14*
*phtP*	*ptx16*	HAD superfamily hydrolase	PSPPH4304	*txi13*	*txi13*
*amtA*	*amtA*	L-arginine:lysine amidinotransferase	PSPPH4303	*txi12*	*txi12*
*phtQ*	*ptx18*	Hypothetical protein	PSPPH4302	*txi11*	*txi11*
*phtS (cysC1*)	*ptx19*	Adenylylsulfate kinase	PSPPH4301	*txi10*	*txi10*
*phtT*	*ptx20*	Hypothetical protein	PSPPH4300	*txi9*	*txi9*
*phtU*	*ptx21*	L-amino acid ligase	PSPPH4299	*txi8*	*txi8*
*phtV*	IS-Sir	ISPsy5 Transposase truncated	PSPPH4298	*txi5*	*txi5*

a Genes annotated in NBCI data base with 100% of coverage and identity with corresponding gene in *Ps* pv. *phaseolicola* NPS3121. Accession numbers of the *P. syringae* strains used in the preparation of this table are as follow: *Ps phaseolicola* 1448A (NC_005773), *Ps phaseolicola* MAFF302282 (AB237164.1) and *Ps actinidiae* Kw11 (AB217563.1).

Several years later, Aguilera *et al.* [49] carried out a functional characterization of the gene cluster involved in phaseolotoxin biosynthesis (the *pht* cluster). It is composed of 23 genes and is flanked by insertion sequences and trasposases, which agrees with the hypothesis of the horizontal transference of the phaseolotoxin cluster. They demonstrated that the mutation of 14 genes within the cluster results in a Tox^−^ phenotype for 11 of them, from *phtA* to *ph*t*T* (Figure 5, Table 2), while three mutants exhibited only low levels of phaseolotoxin production. The Tox^−^ phenotype clearly indicates that all genes within this region encode proteins that are required at one or more of the different stages of phaseolotoxin production, such as synthesis, transport or regulation. The 23 genes are organized in five transcriptional units, two monocistronic and three polycistronic, with one overlapping a larger operon. Thus, *phtL* and *argK* result in two independent monocistronic units, one polycistronic operon comprising 11 genes, from *phtA* to *phtK*, harbouring an internal promoter upstream of *phtD*, and another large operon comprising genes from *phtM* to *phtV* [49] (Figure 5). The authors also observed that null-*phtL* mutants cannot transcribe the *phtM* operon, which suggested a regulatory role for the *phtL* gene product, but this had no effect on any of the other transcriptional units. However, their findings indicate that both the *phtD* and *phtM* operons were transcribed from very similar promoters, suggesting a common mechanism of regulation. This in turn suggested the PhtL did not have an effect on the regulation of the *argK* or *phtA* promoters [49].

After publication of the whole genome sequence of *P. syringae* pv. *phaseolicola* strain 1448A, which was deposited in a database for public access (NC_005773 in the NCBI database), Arai and Kino [50] localized an l-amino acid ligase in the PSPPH_4299 gene in the phaseolotoxin gene cluster. The PSPPH_4299 protein synthesized various hetero-dipeptides, but no homo-dipeptides or tripeptides were detected. It was found that homoarginine was usable as a substrate, but ornithine was not. Further investigation revealed that ATP and Mg^2+^ were essential for peptide synthesis, and that ATP was hydrolysed to ADP and phosphate. Serine, threonine and glycine were also usable as *N*-terminal substrates, and resulted in the synthesis of hetero-dipeptides containing basic amino acids. d-amino acids were not usable as substrates [50]. They concluded that they had found a novel l-amino acid ligase, the PSPPH_4299 protein, which is encoded by a gene in the phaseolotoxin biosynthetic gene cluster from *P. syringae* pv. *phaseolicola* 1448A, suggesting that the l-amino acid ligase plays a physiologically important role in the synthesis of a peptide phytotoxin.

### 3.3. Application of Phaseolotoxin Genes in Disease Diagnosis

The presence of specific genes for phaseolotoxin production gave rise to the development of detection methods like the PCR technique. Although this molecular method can detect strains that produce phaseolotoxin, there are non-toxigenic strains that are still pathogenic. In fact, studies of the *argK-tox* gene cluster in non-toxigenic strains of *P. syringae* pv. *phaseolicola*, which are commonly found in the bean fields of northern Spain, have revealed that these strains are undetectable by available methods of fast detection [51]. PCR analysis of three collections of strains showed the presence of three groups: toxigenic strains with the *phtE* and *argK* sequences, non-toxigenic strains with the *phtE* and *argK* sequences (*argK-tox* cluster) and non-toxigenic strains without the *argK-tox* cluster. González *et al.* [51] revealed that the presence of wild, non-toxigenic strains that cause halo blight but do not carry the *argK-tox* gene cluster represent a drawback to the methods used to detect *P. syringae* pv. *phaseolicola*, as these strains cannot be detected by these methods in dry seeds, and halo blight is spread by sowing contaminated seeds [51].

A similar study was performed by Rico *et al.* [52], who studied the incidence of phaseolotoxin-producing strains in beans with symptoms of disease. Almost 69% of strains of *P. syringae* pv. *phaseolicola* isolated in northern Spain were non-toxigenic. Indeed, the non-toxigenic mutants of *P. syringae* pv. *phaseolicola* were still pathogenic, and their virulence was comparable to that of the wild type, except that there was no formation of chlorotic halos. These results confirm that phaseolotoxin is a virulence factor. They also found that the majority of strains isolated contained *avrPphF*; Thus, they developed an *avr*-PCR method, which extends the range of detection of *P. syringae* pv. *phaseolicola* [52].

These and previous results have led to the conclusion that the *argK-tox* cluster was integrated into the genome of *P. syringae* by horizontal transference. Furthermore, it is possible to detect the *argK-tox* cluster in two pathovars of *P. syringae* (*phaseolicola* and *actinidiae*) that belong to different phylogenetic groups. Therefore, three hypotheses were proposed by Sawada *et al.* [53]: (1) The *argK-tox* gene cluster was introduced by horizontal gene transfer from the original organism into the genome of a common ancestor of pv. *actinidiae* and pv. *phaseolicola*. Afterward, as the ancestor evolved into pv. *actinidiae* and pv. *phaseolicola*, the *argK* gene evolved with the rest of the genome; (2) After pv. *actinidiae* and pv. *phaseolicola* separated from their common ancestor, *argK* was introduced by horizontal gene transfer from the original organism into either pv. *actinidiae* or pv. *phaseolicola*, from which the gene was introduced by a second horizontal gene transfer into the other; (3) After pv. *actinidiae* and pv. *phaseolicola* separated, *argK* was introduced from the original organism into pv. *actinidiae* and pv. *phaseolicola* by two independent horizontal gene transfers. They found that the *argK* gene was introduced very recently, in evolutionary terms, and so rejected hypotheses (1) and (2). Their phylogenetic analysis of the evolutionary mechanism of the *argK-tox* gene cluster showed that it had expanded its distribution over two pathovars by horizontal gene transfer, so the third hypothesis seems to be most likely [53,54]. To confirm the possibility of horizontal transference between an original organism and two different pathovars of *P. syringae*, they performed a comparative analysis of the *argK-tox* cluster and the regions bordering it in phaseolotoxin-producing strains. The authors presented studies which confirmed that the *tox* islands (containing the *argK-tox* cluster, the *phtA-phtH* genes and the *argK* genes) localized on respective chromosomes of pv. *actinidiae* and *phaseolicola* were exogenous DNA regions, and they also clarified that both pathovars were phylogenetically distant from each other. However, almost identical *tox* islands were confirmed to be present on their diversified chromosomes. They identified three coding sequence (CDs) encoding putative tyrosine recombinases at the left end of the *tox* islands. Given that some tyrosine recombinases have been reported to catalyse the integration/excision of genomic islands, the authors hypothesized that these tyrosine recombinases were involved in the horizontal transfer of *tox* islands [55]. These results suggested that the *tox* islands were organized in some unknown bacteria distantly-related to *P. syringae* and then horizontally transferred and integrated site-specifically and in the same direction into the respective homologous sites of the pv. *actinidiae* and pv. *phaseolicola* chromosomes. However, the fact that the relative positions of the *tox* islands on the respective chromosomes of pv. *actinidiae* and pv. *phaseolicola* were quite different implies that the *tox* islands integrated site-specifically into the respective chromosomes after the relative positions of the sites for their integration had changed due to genomic rearrangement [55].

### 3.4. Regulation of Phaseolotoxin Production

The special characteristic of phaseolotoxin is that it can be produced at relatively low temperatures, which has allowed important studies to be conducted on the thermoregulation of genes involved with phaseolotoxin production. In fact, evidence obtained by Rowley *et al.* suggests that a protein produced by *P. syringae* pv. *phaseolicola* at 28 °C that binds specifically to both the thermoregulatory region (TRR) and *argK* fragments could be involved in the negative regulation of the *argK* gene *in vivo* at this temperature [56]. First, motility shift assays showed that there was a protein, detected only in cells grown at 28 °C, that binds specifically to both the TRR and *argK* fragments. Second, cross-competition data showed that the TRR fragment efficiently and specifically competes with the *arg*K fragment for binding of the protein. Third, the sites in the *argK* fragment that were protected from DNAse I cleavage harbour conserved or semi-conserved core motifs found in the TRR [56]. Rowley *et al.* [56] sequenced the largest locus (*phtE*) (Figure 5) in the phaseolotoxin gene cluster; however, this operon is not regulated by temperature. Afterward, the sequences of six additional loci in the toxin cluster were analysed, and the results showed at least two genes harbouring sequences that presented similarity to the core motif [56].

Sometime later, López-López *et al.* [57] proposed a working model for the induction of genes involved in phaseolotoxin synthesis in *P. syringae* pv. *phaseolicola* growing at 18 °C. The signal is processed and transduced to some effector molecule that will act upon the repressor of phaseolotoxin genes. At 28 °C, the genes involved in the synthesis of phaseolotoxin, such as *amtA*, are negatively regulated by a repressor molecule that may bind to TRR motifs; when the temperature is lowered to 16 to 18 °C, the signal is processed and relieves repression mediated by the repressor. These genes are then actively expressed [57]. Analysis of the reverse transcription of *phtL*, the intergenic region of *phtMN* and *amtA*, confirmed that expression of these genes in enhanced by components present in leaf extracts. Additionally, *phtB* and *desI*, which belong to the *phtA* and *phtD* operons, respectively, showed a 1.5-fold increase in expression, values that are statistically significant on the basis of microarray analysis [58]. Recent studies have revealed that the two-component system GacA/GacS controls production of phaseolotoxin. In fact, *gacA*^−^ and *gacS*^−^ mutants in *P. syringae* pv. *phaseolicola* do not produce phaseolotoxin *in vitro* [59]. These results contradict previous reports, in which a *gacS* (*lemA*) mutant was observed that could produce phaseolotoxin [60]; however, the latter results are consistent with numerous data demonstrating the critical role of the highly conserved GacA/GacS system in achieving full virulence and pathogenicity in other pathovars of *P. syringae* [59].

## 4. Mangotoxin

Mangotoxin is a recently discovered antimetabolite toxin produced mainly by *P. syringae* pv. *syringae*. Until now, few other mangotoxin-producing pathovars had been detected, although it would be necessary to check a large number of strains belonging to different pathovars to be conclusive [61]. So far, mangotoxin production has been detected in two strains of *P. syringae*, pv. *avellanea* and pv. *pisi* [21]. Mangotoxin seems to be an oligopeptide [61], and preliminary data suggest that it is composed by of two amino acids linked to a sugar residue (unpublished data). Mangotoxin inhibits ornithine acetyl-transferase (OAT), with inhibitory activity comparable to that produced by the chemical inhibitor p-chloromercuribenzoic acid (PCMB) [61]. Experiments to determine the contribution of mangotoxin production to the severity of disease symptoms and epiphytic fitness were performed using wild-type, toxin-producing strains as well as mutants that are mangotoxin-defective [3]. These experiments revealed that the necrotic symptoms produced by wild-type *P. syringae* pv. *syringae* UMAF0158 (CECT7752) were significantly more extensive and frequent than those produced by mangotoxin-defective mutants, indicating that mangotoxin-defective mutants are less virulent. Therefore, mangotoxin was considered a virulence factor [3,61]. Moreover, similar population densities of mangotoxin-producing strains and defective mutants were detected when hosts were inoculated individually. However, when the wild type UMAF0158 was co-inoculated with a mangotoxin-defective mutant, the mutant had lower population densities. These results suggested that *P. syringae* pv. *syringae* strains producing mangotoxin were more able to colonize the phyllosphere than non-producing strains, even in direct competition [3].

There is still little information available about mangotoxin. Experiments to identify the genes involved in the production of mangotoxin are ongoing. The information that is available comes mainly from analyses of mangotoxin-defective mutants by integration of the mini*Tn5* transposon. The sequence comparison of disrupted genes from several mini*Tn5* mutants showed that, as was observed with both tabtoxin and phaseolotoxin, the global regulator system *gacA*/*gacS* maybe also be involved in the regulation of the mangotoxin production [62]. This regulatory system is composed of two essential genes, *gacA* and *gacS*, encoding a sensor kinase and response regulator, respectively, which control the production of multiple secondary metabolites and are directly related to pathogenesis [63].

**Figure 6:**
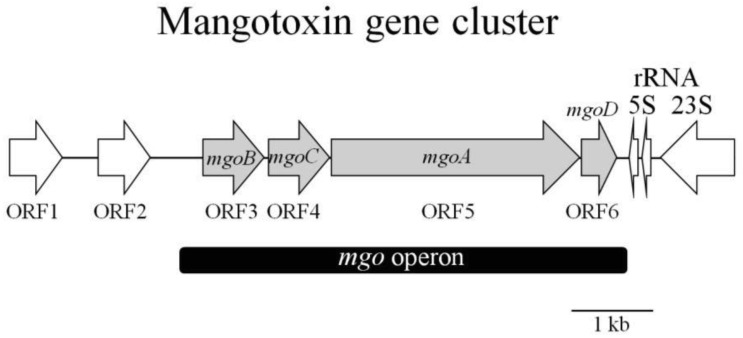
Structural organization of the mangotoxin gene cluster in the genomic clone pCG2-6. The different CDs are represented by arrows indicating the direction of transcription. The grey arrows indicate the *mgo* operon (black band) genes. Other genes identified by researchers are also shown in the figure. Figure partially adapted from Arrebola *et al.* [62].

Other genes involved in mangotoxin production have been detected (Figure 6, Table 3), including *mgoA* [62]. The *mgoA* gene showed high similarity to non-ribosomal peptide synthetase (NRPS), an enzymatic system involved in the synthesis of antibiotic peptides via the non-ribosomal thiotemplate mechanism of biosynthesis. Analysis of the predicted amino acid sequence from *mgoA* indicates that it contains only one amino acid activation module typical of a functional NRPS. It contains the typical aminoacyl adenylation domain, found at the *N*-terminal end, which seems to be responsible for recognizing and adenylating of the carboxylic acid of the amino acid substrate [64]. A condensation domain is conventionally fused to the amino-terminal end of modules accepting acyl groups from the preceding module [65]. Three potential reductase domains were also identified at the carboxyl-terminal end of MgoA [62]. The gene *mgoA* is surrounded by several genes (Figure 6, Table 3), whose functions are still unknown. The genes *mgoB, mgoC* and *mgoD*, corresponding to the ORFs 3, 4 and 6, allow a hypothetical protein group, whose putative function is very difficult to establish because it does not have a clear functional domain. However, a very recent study undertaken to characterize the transcriptional organization of the *mgo* gene cluster has determined the possible functions of *mgoB*, *mgoC* and *mgoD* [66]. In this study, Arrebola *et al.* [66] propose that both *mgoB* and *mgoC* could be oxygenases. Specifically, *mgoB*'*s* product is similar to the Haem oxygenase-like, multi-helical superfamily, and *mgoC* bears significant similarity to the p-aminobenzoate *N*-oxygenase of *Streptomyces thioluteus*. In contrast, *mgoD* is similar to two possible domains, a polyketide cyclase and a lipid transporter domain. These four genes, *mgoBCAD*, are co-transcribed in only one transcript, the absence of which produces mangotoxin-defective mutants. Upstream of *mgoB* is located a functional promoter, where oligonucleotides related to a known transcription factors *rpoD* (σ^70^) binding site are present. A functional terminator has been located downstream of *mgoD* as well, characterizing the complete *mgo* operon [66]. This operon is highly homologous to the *pvf* gene cluster identified in *P. entomophila*, a bacterium that is lethal to *Drosophila melanogaster* [67]. Vallet-Gely *et al.* [67] have proposed that the *pvf* gene cluster of *P. entomophila* could perform a regulatory role involved in the production of virulence factors in *Pseudomonas* species [67]. Based on studies conducted by Vallet-Gely *et al.*, a possible regulatory role for the *mgo* operon could also be proposed. Performing the same experiment described by Vallet-Gely *et al.*, Arrebola *et al.* observed a complementation when extract from wild type UMAF0158 was used in cultures containing mutants for the *gac* and *mgo* operons, which led them to suggest a possible regulatory role for the *mgo* operon similar to that proposed by Vallet-Gely *et al.* for the *pvf* genes [66].

**Table 3:** Mangotoxin genes identified from *Pseudomonas syringae* pv. *syringae* UMAF0158 obtained from the sequencing of the genomic clon pCG2-6 with access NCBI number DQ532441.

**Genes**	**NCBI Protein id**	**Putative function**	**Ortholog genes** a		

			***Ps. syringae* B728a**	***Ps. tomato*** **DC3000**	***Ps. phaseolicola*** **1448A**
ORF1	ABG00042.1	aldo/keto reductase	Psyr5007	PSPTO5453	PSPPH5086
ORF2	ABG00043.1	regulatory protein GntR	Psyr5008	PSPTO5454	PSPPH5087
ORF3 (*mgoB*)	ABG00044.1	Hypothetical protein	Psyr5009	PSPTO5455	PSPPH5088
ORF4 (*mgoC*)	ABG00045.1	Hypothetical protein	Psyr5010	PSPTO5456	PSPPH5089
ORF5 (*mgoA*)	ABG00046.1	non-ribosomal peptide synthetase	Psyr5011	PSPTO5457	PSPPH5090
ORF6 (*mgoD*)	ABG00047.1	Hypothetical protein	Psyr5012	PSPTO5458	PSPPH5091

a Genes annotated in NBCI data base with 100% of coverage and identity with corresponding gene in *Ps* pv. *syringae* UMAF0158 (CECT7752). Accession numbers of the *P. syringae* strains used in the preparation of this table are as follow: *Ps syringae* B728a (CP000075), *Ps tomato* DC3000 (AE16853.1) *Ps phaseolicola* 1448A (NC_005773).

At the same time, these authors also obtained evidence of other genes, absent in the genome of *P. syringae* pv. *syringae* B728a, which could also be involved in specific processes for the production of mangotoxin, suggesting specific reactions for mangotoxin production encoded out of the predicted *mgo* operon containing the putative NRPS of *mgoA* [62]. These specific genes were located in the genome of the wild type *P. syringae* pv. *syringae* UMAF0158 and present an operon-like structure (unpublished data). This cluster consists of six ORFs that have been given the acronym *mbo*, for mangotoxin biosynthetic operon, and each ORF has been ordered from A to F. The mutation of this cluster results in mangotoxin-defective mutants, and the most surprising result is that when theses *mbo* genes are introduced into *P. syringae* pv. *syringae* B728a on a plasmid, this non-producing strain turns into a mangotoxin-producing strain [67]. For this reason, Carrión *et al.* [67] have attributed this biosynthetic capacity to the *mbo* operon. The specificity of these genes makes it possible to develop a PCR-based detection method for mangotoxin producing strains [67], providing easy identification for these kind of strains in field samples (unpublished data).

## 5. Conclusions

After compilation of the information discussed in this review, it is possible to conclude that *Pseudomonas syringae* is a bacterial species that is perfectly adapted to the plant environment, and an important part of this adaptation is the production of wide range of virulence factors. The antimetabolite toxins are a group of small, peptidic molecules composed of a few amino acids linked to another chemical structure, which can act as inhibitors of key enzymes in the nitrogen metabolism of the plant host. These toxins seem not to be exclusive to any particular pathovar or plant host, but neither do they seem to be produced by other phytopathogenic species. From the genetic point of view, the biosynthesis of this kind of toxin is related to non-ribosomal synthesis, where the biosynthetic genes are grouped in a gene cluster, and all the enzymes required for the assembly and maturation of the toxin, even those needed for secretion of it, are located together [43]. These clusters can be located in genomic islands that can be transferred horizontally between strains of different pathovars [55]. This is in accordance with the non-specific production of toxins in the *P. syringae* pathovars. All of the genomic islands can be regulated by the *gacA*/*gacS* system, which is also in accordance with the hypothesis of horizontal transfer, and the regulation of antimetabolite toxin production has to be accomplished by a common regulation system to prevent excessive production. Finally, the presence of genes exclusive to the antimetabolite toxins have allowed researchers to develop molecular techniques to detect toxin-producing strains related with important diseases, making possible the early detection and control of diseases.

## References

[1] Bender C., Alarcón-Chaidez F., Gross D. (1999). *Pseudomonas syringae* phytotoxins: mode of action, regulation, and biosynthesis by peptide and polyketide synthetases. Microbiol. Mol. Biol. Rev..

[2] Mitchell R.E. (1984). The relevance of non-host toxins in the expression of virulence by pathogens. Annu. Rev. Phytopathol..

[3] Arrebola E., Cazorla F.M., Codina J.C., Gutierrez-Barranquero J.A., Pérez-García A., de Vicente A. (2009). Contribution of mangotoxin to the virulence and epiphytic fitness of *Pseudomonas syringae* pv. *syringae*. Int. Microbiol..

[4] Iacobellis N.S., Lavermicocca P., Surico G., Durbin R.D. (1992). The occurrence and characterization of a syringomycin-macromolecular complex in cultures of *Pseudomonas syringae* pv. *syringae*. Physiol. Mol. Plant Pathol..

[5] Raaijmakers J.M., de Brujin I., de Kock J.D. (2006). Cyclic lipopeptide production by plant-associated *Pseudomonas spp.*: diversity, activity, biosynthesis, and regulation. Mol. Plant Microbe Interact..

[6] Scholz-Schroeder B.K., Soule J.D., Gross D.C. (2003). The *sypA*, *sypB*, and *sypC* synthetase genes encode twenty-two modules involved in the nonribosomal peptide synthesis of syringopeptin by *Pseudomonas syringae* pv. *syringae* strain B301D. Mol. Plant Microbe Interact..

[7] Gross H, Loper J.E. (2009). Genomics of secondary metabolite production by *Pseudomonas* spp.. Nat. Prod. Rep..

[8] Melotto M., Underwood W., Koczan J., Nomura K., He S.Y. (2006). Plant stomata function in innate immunity against bacterial invasion. Cell.

[9] Brooks D.M., Bender C.L., Kunkel B.N. (2005). The *Pseudomonas syringae* phytotoxin coronatine promotes virulence by overcoming salicylic acid-dependent defenses in *Arabidopsis thaliana*. Mol. Plant Pathol..

[10] Uppalapati S.R., Ishiga Y., Wangdi T., Kunkel B.N., Anand A., Mysore K.S., Bender C.L. (2007). The phytotoxin coronatine contribute to pathogen fitness and is required for suppression of salicylic acid accumulation in tomato inoculated with *Pseudomonas syringae* pv. *tomato* DC3000. Mol. Plant Microbe Interact..

[11] Rangaswamy V., Bender C.L. (2000). Phosphorylation of CorS and CorR, regulatory proteins that modulate production of the phytotoxin coronatine in *Pseudomonas syringae*. FEMS Microbiol. Lett..

[12] Parry R.J., Lin M.T., Walker A.E., Mhaskar S. (1991). Biosynthesis of coronatine: investigations of the biosynthesis of coronamic acid. J. Am. Chem. Soc..

[13] Parry R.J., Mhaskar S., Lin M.T., Walker A.E., Mafoti R. (1994). Investigations of the biosynthesis of the phytotoxin coronatine. Can. J. Chem..

[14] Penfold C.N., Bender C.L., Turner J.G. (1996). Characterisation of genes involved in biosynthesis of coronafacic acid, the polyketide component of the phytotoxin coronatine. Gene.

[15] Slawiak M., Lojkowska E. (2009). Genes responsible for coronatine synthesis in *Pseudomonas syringae* present in the genome of soft rot bacteria. Eur. J. Plant Pathol..

[16] Mitchell R.E. (1976). Isolation and structure of a chlorosis inducing toxin of *Pseudomonas phaseolicola*. Phytochemistry.

[17] Turner J.G., Debbage J.M. (1982). Tabtoxin-induced symptoms are associated with the accumulation of ammonia formed during photorespiration. Physiol. Plant Pathol..

[18] Cazorla F.M., Olalla L., Torés J.A., Codina J.C., Pérez-García A., de Vicente A., Rudolf K., Burr T.J., Mansfield J.W., Stead D., Vivian A., von Kietzell J. (1997). *Pseudomonas syringae* pv. *syringae* as microorganism involved in apical necrosis of mango: characterization of some virulence factors. Pseudomonas syringae Pathovars and Related Species.

[19] Völksch B., Weingart H. (1998). Toxin production by pathovars of *Pseudomonas syringae* and their antagonistic activities against epiphytic microorganism. J. Basic Microbiol..

[20] Hwang M.S.H., Morgan R.L., Sarkar S.F., Wang P.W., Guttman D.S. (2005). Phylogenetic characterization of virulence and resistance phenotypes of *Pseudomonas syringae*. Appl. Environ. Microbiol..

[21] Murillo J., Bardaji L., Navarro de la Fuente L., Führer M.E., Aguilera S., Álvarez-Morales A. (2011). Variation in conservation of the cluster for biosynthesis of the phytotoxin phaseolotoxin in *Pseudomonas syringae* suggest at least two events of horizontal acquisition. Res. Microbiol..

[22] Durbin R.D., Uchytil T.F. (1985). Role of zinc in regulating tabtoxin production. Experientia.

[23] Stewart W.W. (1971). Isolation and proof of structure of wildfire toxin. Nature.

[24] Uchytil T.F., Durbin R.D. (1980). Hydrolysis of tabtoxin by plant and bacterial enzymes. Experienta.

[25] Manning J.M., Moore S., Rowe W.B., Meister A. (1969). Identification of l-methionine s-sulfoximine as the diastereoisomer of l-methionine SR-sulfoximine that inhibits glutamine synthetase. Biochemistry.

[26] Anzai H., Yoneyama K., Yamaguchi I. (1989). Transgenic tobacco resistant to a bacterial disease by the detoxification of a pathogenic toxin. Mol. Gen. Genet..

[27] Murakami T., Anzai H., Imai S., Satoh A., Nagaoka K., Thompson C.J. (1986). The bialaphos biosynthetic genes of *Streptomyces hygroscopicus*: molecular cloning and characterization of the gene cluster. Mol. Gen. Genet..

[28] Thompson C.J., Movva N.R., Tizard R., Crameri R., Davies J.E., Lauwereys M., Botterman J. (1987). Characterization of herbicide-resistance gene bar from *Streptomyces hygroscopicus*. EMBO J..

[29] He H., Ding Y., Bartlam M., Sun F., Le Y., Qin X., Tang H., Zhang R., Joachimiak A., Liu J., Zhao N., Rao Z. (2003). Crystal structure of tabtoxin resistance protein complexed with acetyl coenzyme A reveals the mechanism for β-lactam acetylation. J. Mol. Biol..

[30] Kinscherf T.G., Coleman R.H., Barta T.M., Willis D.K. (1991). Cloning and expression of the tabtoxin biosynthesis region from *Pseudomonas syringae*. J. Bacteriol..

[31] Willis D.K., Holmstadt J.J., Kinscherf T.G. (2001). Genetic evidence that loss of virulence associated with gacS mutation in Pseudomonas syringae B728a does not result from effects on alginate production. App. Environ. Microbiol..

[32] Barta T.M., Kinscherf T.G., Willis D.K. (1992). Regulation of tabtoxin production by the lemA gene in *Pseudomonas syringae*. J. Bacteriol..

[33] Barta T.M., Kinscherf T.G., Uchytil T.F., Willis D.K. (1993). DNA sequence and transcriptional analysis of the tblA gene required for tabtoxin biosynthesis by *Pseudomonas syringae*. Appl. Environ. Microbiol..

[34] Liu L., Shaw P.D. (1997). Characterization of *dapB*, a gene required by *Pseudomonas syringae* pv. *tabaci* BR2.024 for lysine and tabtoxinine-β-lactam biosynthesis. J. Bacteriol..

[35] Kinscherf T.G., Willis D.K. (2005). The biosynthetic gene cluster for the β-lactam antibiotic tabtoxin in *Pseudomonas syringae*. J. Antibiot..

[36] Klockgether J., Reva O., Larbig K., Tummler B. (2004). Sequence analysis of the mobile genome island pKLC102 *of Pseudomonas aeruginosa* C. J. Bacteriol..

[37] Bachmann B.O., Li R., Townsend C.A. (1998). Beta-lactam synthetase: a new biosynthetic enzyme. Proc. Natl. Acad. Sci. USA.

[38] Lydon J, Patterson C.D. (2001). Detection of tabtoxin-producing strains of *Pseudomonas syringae* by PCR. Lett. Appl. Microbiol..

[39] Moore R.E., Niemczura W.P., Kwok O.C.H., Patil S.S. (1984). Inhibition of ornithine carbamoyltransferase from *Pseudomonas syringae* pv. *phaseolicola*. Revised structure of phaseolotoxin. Tetrahedron Lett..

[40] Tourte C., Manceau C. (1995). A strain of *Pseudomonas syringae* which does not belong to pathovar *phaseolicola* produces phaseolotoxin. Eur. J. Plant Pathol..

[41] Peet R.C., Lindgren P.B., Willis D.K., Panopoulos N.J. (1986). Identification and cloning of genes involved in phaseolotoxin production by *Pseudomonas syringae* pv. *phaseolicola*. J. Bacteriol..

[42] Hatziloukas E., Panopoulos N.J. (1992). Origin, structure, and regulation of *argK*, encoding the phaseolotoxin-resistant ornithine carbamoyltransferase in *Pseudomonas syringae* pv. phaseolicola, and functional expression of *arg*K in transgenic tobacco. J. Bacteriol..

[43] Zhang Y., Rowley K.B., Patil S.S. (1993). Genetic organization of a cluster of genes involved in the production of phaseolotoxin, a toxin produced by *Pseudomonas syringae* pv. phaseolicola. J. Bacteriol..

[44] Rowley K.B., Clements D.E., Mandel M., Humphreys T., Patil S.S. (1993). Multiple copies of a DNA sequence from *Pseudomonas syringae* pathovar phaseolicola abolish thermoregulation of phaseolotoxin peoduction. Mol. Microbiol..

[45] Hatziloukas E., Panopoulos N.J., Delis S., Prosen D.E., Schaad N.W. (1995). An open reading frame in 28 kb phaseolotoxin gene cluster encodes a polypeptide with homology to fatty acid desaturases. Gene.

[46] Reuter G., Bushell M.E., Gräfe U. (1989). Enzymatic regulation of microbial phytoeffector biosynthesis. Bioactive Metabolites from Microorganisms.

[47] Markisch U., Reuter G. (1990). Biosynthesis of homoarginine and ornithine as precursors of the phytoeffector phaseolotoxin by the amidinotransferase in *Pseudomonas syringae* pv. phaseolicola. J. Basic Microbiol..

[48] Hernández-Guzmán G., Álvarez-Morales A. (2001). Isolation and characterization of the gene coding for the amidinotransferase involved in the biosynthesis of phaseolotoxin in *Pseudomonas syringae* pv. phaseolicola. Mol. Plant Microbe Interact..

[49] Aguilera S., López-López K., Nieto Y., Garcidueñas-Piña R., Hernández-Guzmán G., Hernández-Flores J.L., Murillo J., Álvarez-Morales A. (2007). Functional characterization of the gene cluster from *Pseudomonas syringae* pv. *phaseolicola* NPS3121 involved in synthesis of phaseolotoxin. J. Bacteriol..

[50] Arai T., Kino K. (2008). A novel l-amino acid ligase is encode by a gene in the phaseolotoxin biosynthetic gene cluster from *Pseudomonas syringae* pv. *phaseolicola* 1448A. Biosci. Biotechnol. Biochem..

[51] González A.I., Pérez de la Vega M., Ruiz M.L., Polanco C. (2003). Analysis of the *arg*K-*tox* gene cluster in nontoxigenic strains of *P. syringae* pv. *Phaseolicola*. Appl. Environ. Microbiol..

[52] Rico A., López R., Asensio C., Aizpún M.T., Asensio M.C., Manzanera S., Murillo J. (2003). Nontoxigenic strains of *P. syringae* pv. *phaseolicola* are a main cause of halo blight of vean in Spain and escape current detection methods. Bacteriology.

[53] Sawada H., Suzuki F., Matsuda I., Saitou N. (1999). phylogenetic analysis of *Pseudomonas syringae* pathovars suggest the horizontal gene transfer of *arg*K and the evolutionary stability of *hrp* gene cluster. J. Mol. Evol..

[54] Sawada H., Kanaya S., Tsuda M., Suzuki F., Azegami K., Saitou N. (2002). A phylogenomic study of the OCTase genes in *Pseudomonas syringae* pathovars: the horizontal transfer of the *arg*K-*tox* cluster and the evolucionary history of OCTase genes on their genomes. J. Mol. Evol..

[55] Genka H., Baba T., Tsuda M., Kanaya S., Mori H., Yoshida T., Noguchi M.T., Sawada H. (2006). Comparative analysis of *arg*K-*tox* cluster and their flanking region in phaseolotoxin-producing *Pseudomonas syringae* pathovars. J. Mol. Evol..

[56] Rowley K.B., Xu R., Patil S.S. (2000). Molecular analysis of thermoregulation of phaseolotoxin resistant ornithine carbamoyltransferase (*arg*K) from *Pseudomonas syringae* pv. *phaseolicola*. Mol. Plant Microbe Interact..

[57] López-López K., Hernández-Flores J.L., Cruz-Aguilar M., Álvarez-Morales A. (2004). In *Pseudomonas syringae* pv. *phaseolicola*, expression of the *arg*K gene, encoding the phaseolotoxin resistant ornithine carbamoyltransferase, is regulated indirectly by temperature and directly by a precursor resembling carbamoylphosphate. J. Bacteriol..

[58] Hernández-Morales A., De la Torre-Zavala S., Ibarra-Laclette E., Hernández-Flores J.L., Jofre-Garfias A.E., Martínez-Antonio A., Álvarez-Morales A. (2009). Transcriptional profile of *Pseudomonas syringae* pv. *phaseolicola* NPS3121 in response to tissue extracts from a susceptible *Phaseolus vulgaris* L. cultivar. BMC Microbiol..

[59] De la Torre-Zavala S., Aguilera S., Ibarra-Laclete E., Hernández-Flores J.L., Hernández-Morales A., Murillo J., Álvarez-Morales A. (2011). Gene expression of Pht cluster genes and a putative non-ribosomal peptide synthetase required for phaseolotoxin production is regulated by GacS/GacA in *Pseudomonas syringae* pv. phaseolicola. Res. Microbiol..

[60] Rich J.J., Hirano S.S., Willis D.K. (1992). Pathovar-specific requirement for the *Pseudomonas syringae lem*A gene in disease lesion formation. Appl. Environ. Microbiol..

[61] Arrebola E., Cazorla F.M., Durán V.E., Rivera E., Olea F., Codina J.C., Pérez-García A., de Vicente A. (2003). Mangotoxin: a novel antimetabolite toxin produced by *Pseudomonas syringae* inhibiting ornithine/arginine biosynthesis. Physiol. Mol. Plant Pathol..

[62] Arrebola E., Cazorla F.M., Romero D., Pérez-García A., de Vicente A. (2007). A nonrobosomal peptide synthetase gene (*mgo*A) of *Pseudomonas syringae* pv. *syringae* is involved in mangotoxin biosynthesis and is required for full virulence. Mol. Plant Microbe Interact..

[63] Kim Y.C., Leveau J., McSpadden-Gardener B.B., Pierson E.A., Pierson L.S., Ryu C.M. (2011). The multifactorial basis for plant health promotion by plant-associated bacteria. App. Environ. Microbiol..

[64] Challis G.L., Ravel J., Townsend C.A. (2000). Predictive, structure based model of amino acid recognition by nonribosomal peptide synthetase adenylation domains. Chem. Biol..

[65] Konz D., Marahiel M.A. (1999). How do peptide synthetases generate structural diversity?. Chem. Biol..

[66] Arrebola E., Carrión V.J., Murillo J., de Vicente A., Cazorla F.M. Unravelling molecular aspects of mangotoxin, a virulence factor produced by the phytopathogenic strain *Pseudomonas syringae* pv. *syringae* UMAF0158.

[67] Vallet-Gely I., Opota O., Bonafice A., Novikov A., Lemaitre B. (2010). A secondary metabolite acting as a signalling molecule controls *Pseudomonas entomophila* virulence. Cell. Microbiol..

[67] Carrión V.J., Arrebola E., Cazorla F.M., Murillo J., de Vicente A. Analysis of a *Pseudomonas syringae* operon involved in mangotoxin biosynthesis.

